# PLODs: Novel prognostic biomarkers and potential immunotherapy targets for head and neck squamous cell carcinoma

**DOI:** 10.1016/j.heliyon.2023.e13479

**Published:** 2023-02-03

**Authors:** Siming Gong, Changwu Wu, Yingjuan Duan, Jinfei Fu, Yuling Wang, Hao Wu, Bixi Zhang, Juyu Tang, Panfeng Wu

**Affiliations:** a Department of Hand and Microsurgery, Department of Orthopedics, Xiangya Hospital of Central South University, Changsha, China; b Institute of Anatomy, University of Leipzig, Leipzig, Germany; c Faculty of Chemistry and Mineralogy, University of Leipzig, Leipzig, Germany; d Department of Gastroenterology, Third Xiangya Hospital, Central South University, Changsha, China; e Department of Pathology, Hunan Provincial People's Hospital, Hunan Normal University, Changsha, China

**Keywords:** Head and neck squamous cell carcinoma, PLODs, Prognostic, Tumor microenvironment, Immune infiltration, Immunotherapy

## Abstract

Head and neck squamous cell carcinoma (HNSCC) comprise a group of malignant tumors arising from the squamous epithelium of the oral cavity, pharynx, and larynx. HNSCC is the 6th most common cancer in the world, with approximately 650,000 new cases and 400,000 deaths annually. Although survival rates have improved, HNSCC therapy may result in short – or long-term morbidity in approximately 50% of cases. Previous studies have also indicated that the overexpression of procollagen-Lysine, 2-Oxoglutarate 5-Dioxygenases (PLOD) family proteins could lead to certain diseases or even tumors. However, there has been no dedicated evaluation of the relationship between PLOD family members and HNSCC. Here we used data from the Cancer Genome Atlas (TCGA), Genotype-Tissue Expression (GTEx), and Human Protein Atlas (HPA) databases to explore the potential role of PLOD family proteins in HNSCC. Our evaluations suggest that increased expression of PLOD family proteins may be associated with poorer prognosis and increased immune infiltration in HNSCC, making these proteins a potential biomarker for personalized treatment of HNSCC.

## 1 Introduction

Head and neck squamous cell carcinoma (HNSCC) are a common term used to describe a group of malignant tumors arising from the squamous epithelium of the oral cavity, pharynx, or larynx [1]. Previous studies have shown that HNSCC is the 6th most common cancer in the world with approximately 650,000 new cases and 400,000 deaths per year [2]. Global evaluations suggest that the consumption of tobacco and alcohol products are the most common factor contributing to the development of HNSCC but betel nut chewing has been uniquely identified as an independent factor for HNSCC tumor production in some regions, including various Asian countries [[3], [4], [5], [6]]. Moreover, infection with human papillomavirus (HPV) can contribute to the rising number of HNSCC cases, and while survival rates have improved, HNSCC therapy may result in short-term or long-term morbidity in approximately 50% of patients [7,8]. Various treatments have been applied to HNSCC, including radiotherapy, drug therapy, and surgical resection. However, toxicity following radiotherapy and the challenges around reconstruction after surgery limit these approaches [[9], [10], [11]]. This means that progress in new drug development is rapid. The first molecular-targeting drug, cetuximab, has been shown to be reasonably effective in treating HNSCC when applied in conjunction with radiotherapy or cytotoxic drug therapy [12,13]. Pembrolizumab monotherapy was adopted as the first-line treatment for HNSCC in 2019 with moderate efficacy [14]. Thus, molecular-targeting drugs could be beneficial for patients with HNSCC. Therefore, there is an urgent need to understand the molecular mechanisms and oncogenesis of HNSCC as well as to identify robust biomarkers for therapeutic evaluation.

Recent advances have cemented the fact that the tumor microenvironment (TME) plays a crucial role in tumor formation, drug response, and immune infiltration [15]. The extracellular matrix (ECM) is the major component of the TME, and collagen is an indispensable element in the ECM. Given this, abnormal collagen deposition and production can lead to many diseases and in some extreme cases carcinoma formation [16,17]. These carcinomas form and proliferate based on the deposition and cross-linking of abnormal collagen [17]. Lysyl hydroxylase (LH) is necessary for collagen biosynthesis and is encoded by procollagen-Lysine, 2-Oxoglutarate 5-Dioxygenase (PLOD). The PLOD family includes PLOD1, PLOD2, and PLOD3, all of which may be linked to various cancers, including gastric cancer, sarcoma, and liver cancer [[18], [19], [20]]. Our previous work reported that PLOD3 likely acts as an oncogene, and overexpression of this protein may lead to various tumors [21]. In addition, changes in the expression of the PLOD family have been linked to lower grade glioma (LGG) and soft tissue sarcoma and could be a regulator of the TME [22,23]. Given this, we designed this study to focus on the prognostic function and molecular mechanisms of the PLOD family in HNSCC.

## 2 Materials and methods

### 2.1 ONCOMINE analysis

The ONCOMINE tool is a user-friendly online tool designed to produce high-quality data analysis. Given this we used ONCOMINE to evaluate the expression of various PLODs from various tumors using the following parameters: p < 0.05; fold change >2; Type of analysis: cancers vs. normal; level of the data: mRNA.

### 2.2 GEPIA2 analysis

We then used gene expression profiling interactive analysis 2 (GEPIA2) tool to evaluate the expression of various PLODs in HNSCC tumor tissues and their corresponding normal tissues using the data from various TCGA and GTEx cohorts. We then used the “Expression DIY” kit from the “Expression Analysis” model to identify differential PLOD expression in HNSCC samples using the following parameters: p value “0.01,” Matched Normal data “Match TCGA normal and GTEx data,” Tissue Order: “HNSC”. We also used GEPIA2 to identify the top 100 PLOD-related genes which indicate the genes that has a similar expression pattern with PLOD genes in HNSCC.

### 2.3 GEO database analysis

Gene Expression Omnibus (GEO) database was employed to validate the results from TCGA and GTEx database. In the present study, GSE29330, GSE55549 and GSE23036 were used to achieve the validation [[24], [25], [26]]. P < 0.05 and fold change >2 would be viewed as statistical significance.

### 2.4 cBioPortal analysis

The cBioPortal tool was used to obtain PLOD gene alteration data based on the TCGA dataset. All PLOD family members were evaluated using the HNSCC pan-cancer atlas (TCGA) cohort. The names of the PLOD genes (PLOD1, PLOD2 and PLOD3) were typed into the box and then the genetic alteration data was then obtained from both the “OncoPrint” and “Cancer Types Summary” models provided by cBioportal.

### 2.5 Datasets

RNA-sequencing data and clinical information for various HNSCC patients were obtained using the Xena tool. All samples from the TCGA dataset were included for further analysis (Supplementary Table S1) and the data were normalized and log2 (x+1) transformed.

### 2.6 Survival analysis

After transformation we used R software (version 3.6.3) to analyze the relationship between PLOD expression and HNSCC prognosis. We then used R packages “survminer” and “survival” to complete the survival Kaplan-Meier plots. The optimal evaluation cut-off was determined separately using statistical significance [27]. Moreover, the survival impact of PLOD expression was also analyzed using the PLOD expression as a continuous variable.

### 2.7 Immune infiltration analysis

R package “GSVA” was then used to produce the single sample gene set enrichment analysis (ssGSEA) and facilitate the relative immune cell quantitation for these datasets [28]. R package “ESTIMATE” was then applied to calculate the ImmuneScore (correlated with the level of immune cell infiltration), StromalScore (correlated with the level of stroma cell) and ESTIMATEScore (negatively correlated with tumor purity) [29]. In this study, the patients were separated averagely to analyze the ImmuneScore, StromalScore and ESTIMATEScore (cut-off: PLOD1 = 6.72, PLOD2 = 4.89, PLOD3 = 5.39). The TIMER tool was then used to assess immune cell infiltration in HNSCC patients.

### 2.8 Gene set enrichment analysis

R package “clusterProfiler” (version 3.14.3) was then used to complete the Gene set enrichment analysis (GSEA) based on gene set c2. cp.v7.2. symbols.gmt. The significance threshold was set at p < 0.05, and the false discovery rate (FDR) was set to < 0.25.

## 3 Results

### 3.1 Expression of PLOD family members in HNSCC

**Fig. 1:**
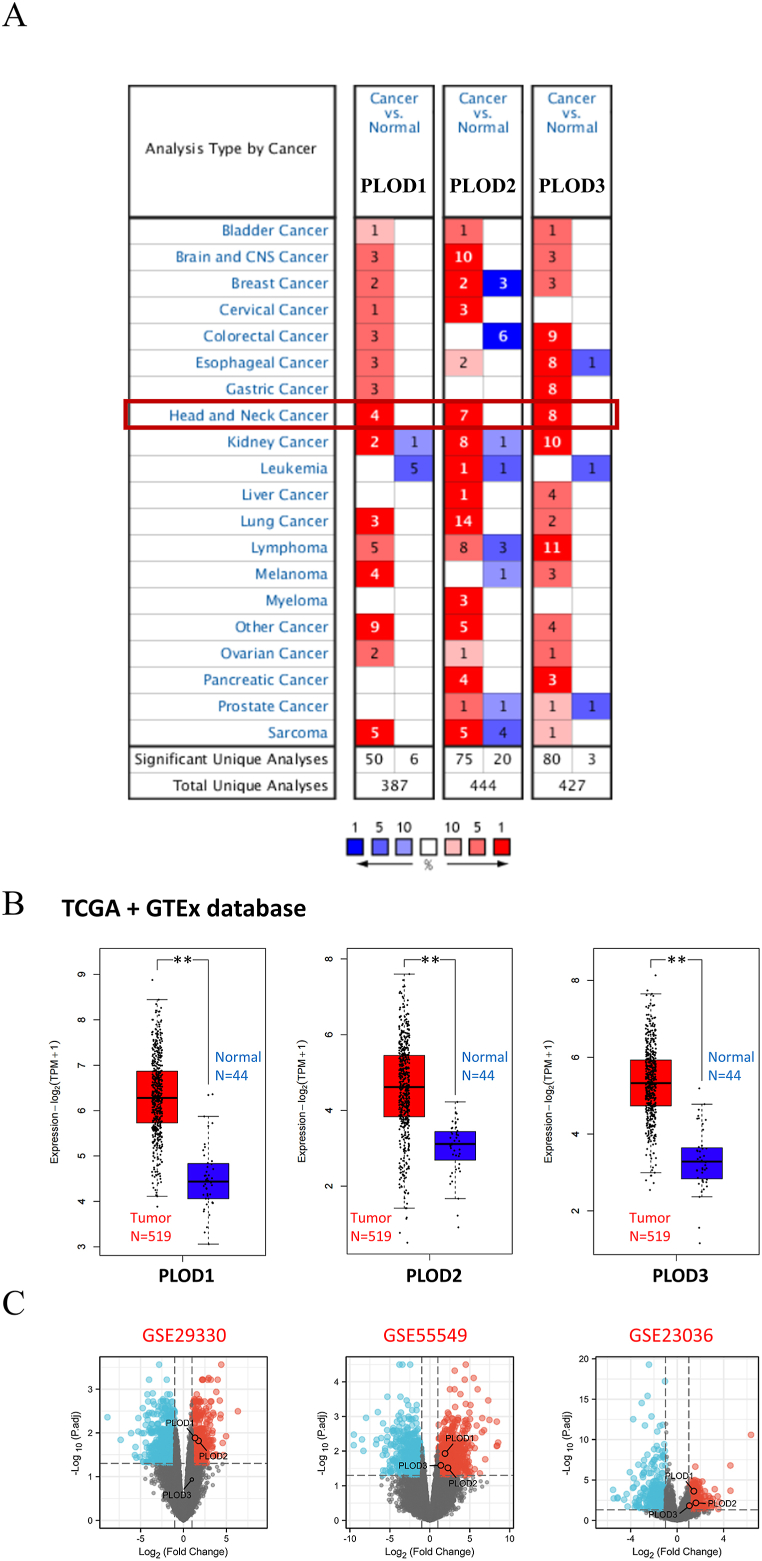
PLOD expression analysis. **(A)** The expression of PLODs across different types of cancers based on ONCOMINE evaluations. (**B**) The expression of PLODs in HNSCC. **(C)** The PLOD expression in GEO database (GSE29330, GSE55549 and GSE23036). **p < 0.01.

The expression of the PLOD family members in HNSCC was determined using ONCOMINE analysis. The PLOD family members (PLOD1, PLOD2, and PLOD3) were overexpressed in HNSCC tumor tissue (Fig. 1A). The HNSCC dataset produced from a combination of TCGA database and corresponding normal tissues from the GTEx cohort were also analyzed using GEPIA2. These evaluations indicated that PLOD1, PLOD2, and PLOD3 (Fig. 1B) might be high expressed in HNSCC tumor tissues compared with normal tissues (all p < 0.01). Addtionally, results from GEO database also indicate that the PLOD family members could be overexpressed in tumor tissue (Fig. 1C).

### 3.2 Survival analysis in response to PLOD expression

**Fig. 2:**
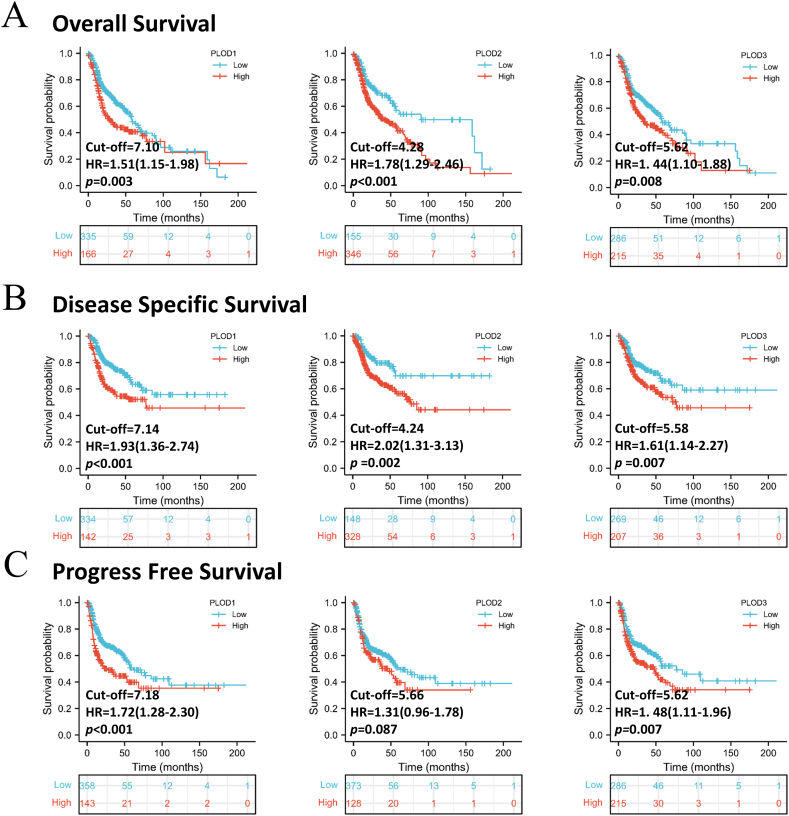
Correlation between PLOD expression and survival prognosis in HNSCC. Figure summarizes three types of survival analysis including **(A)** overall survival; **(B)** disease specific survival; (**C**) progression free survival.

Survival analysis was completed using R software and the TCGA database samples. We completed three different types of survival analyses in order to explore the relationship between the expression of these PLODs and HNSCC prognosis. In both the overall (OS) and disease specific survival (DSS) analyses, PLOD expression was shown to be closely associated with poor prognosis in HNSCC with these evaluations determining a hazard ratio range of 1.51 (PLOD1) to 1.78 (PLOD2) (Fig. 2A and B, all p < 0.01). Progression free survival (PFS) analysis revealed that both PLOD1 and PLOD3 were linked to poor prognosis in HNSCC (both p < 0.01), while there was no significant association between PFS and PLOD2 (Fig. 2C, p = 0.087). The 1-, 3- and 5-years of survival rates at the same cut-off were also obtained (Supplementary Table S2). Additionally, the survival analysis using the PLOD expression as a continuous variable was also obtained (Supplementary Table S3). The patients with high-expression of PLOD seems to have a low 5-years survival but the Cox regression analysis indicated that PLOD3 is not associated with prognosis.

### 3.3 Alterations to PLOD and related-gene analysis in HNSCC

**Fig. 3:**
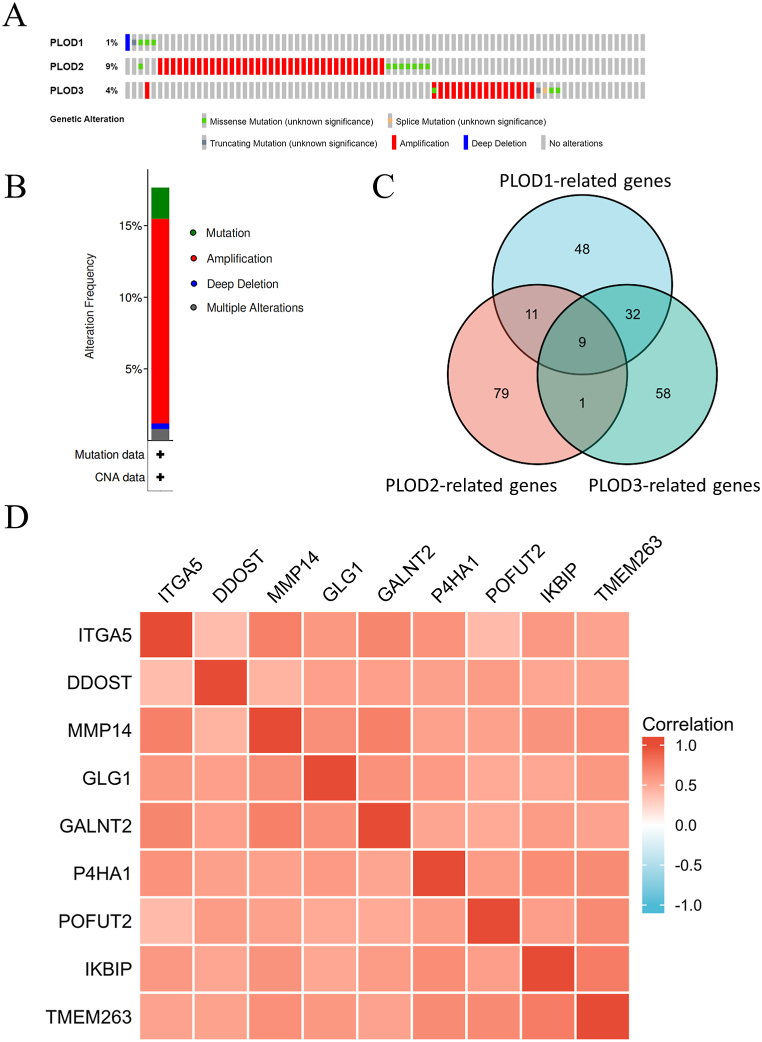
Evaluating genetic alterations in the PLODs and PLOD-related genes. **(A)** Correlation between changes in the frequencies of specific alterations and the type of PLOD (PLOD1, PLOD2 and PLOD3) expressed in each tissue sample. **(B)** Mutational analysis of all PLODs. **(C)** Intersection analysis for PLOD-related genes. **(D)** Identification of nine co-expressing genes associated with various PLOD expression (PLOD1, PLOD2 and PLOD3).

The PLOD genetic alteration data for HNSCC were obtained from cBioPorta. Evaluation of this data revealed that PLOD2 and PLOD3 mutations most commonly presented as amplifications of these genes, 7.06% and 3.23%, respectively. While mutation accounted for most of the alterations in PLOD1 (0.81%) (Fig. 3A). Overall, amplification (10.08%) was the most common type of genetic alteration, followed by mutation (2.82%) in HNSCC (Fig. 3B). In addition, our analysis identified nine genes that were closely associated with all three PLODs (PLOD1, PLOD2, and PLOD3) based on the PLOD-related genes (Fig. 3C, Supplementary Table S4). Co-expression of these nine genes, namely integrin, alpha 5 (ITGA5), dolichyl-diphosphooligosaccharide-protein glycosyltransferase non-catalytic subunit (DDOST), matrix metallopeptidase 14 (MMP14), Golgi glycoprotein 1 (GLG1), polypeptide *N*-acetylgalactosaminyltransferase 2 (GALNT2), Prolyl 4-Hydroxylase Subunit alpha 1 (P4HA1), protein *O*-fucosyltransferase 2 (POFUT2), inhibitor of nuclear factor kappa-B kinase interacting protein (IKBIP), and transmembrane protein 263 (TMEM263) was verified using R software and all nine genes were positively correlated with each other (Fig. 3D).

### 3.4 PLOD expression and immune infiltration in HNSCC

**Fig. 4:**
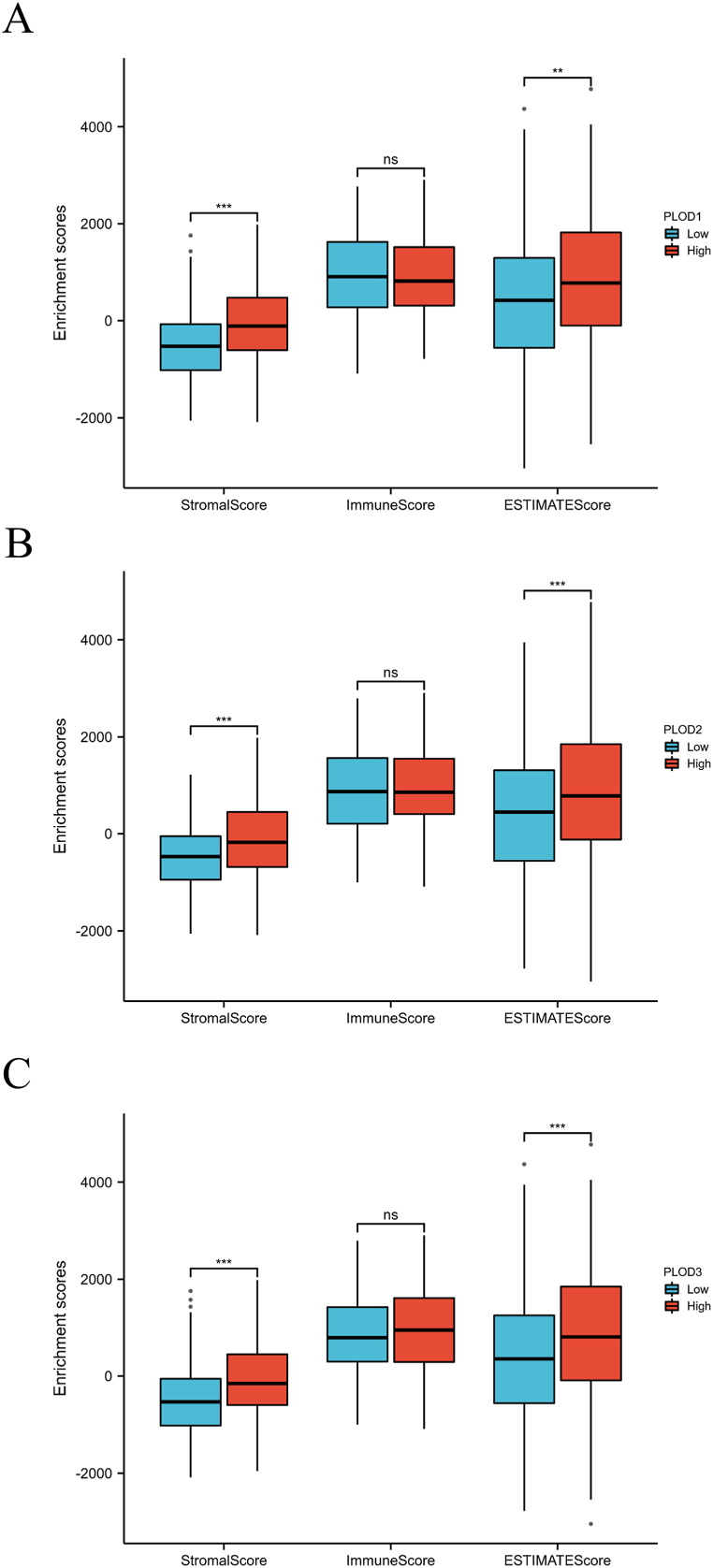
Correlation between PLOD expression and ImmuneScore, StromalScore and ESTIMATEScore. (A) PLOD1; (B) PLOD2; (C) PLOD3. **p < 0.01 ***p < 0.001.

**Fig. 5:**
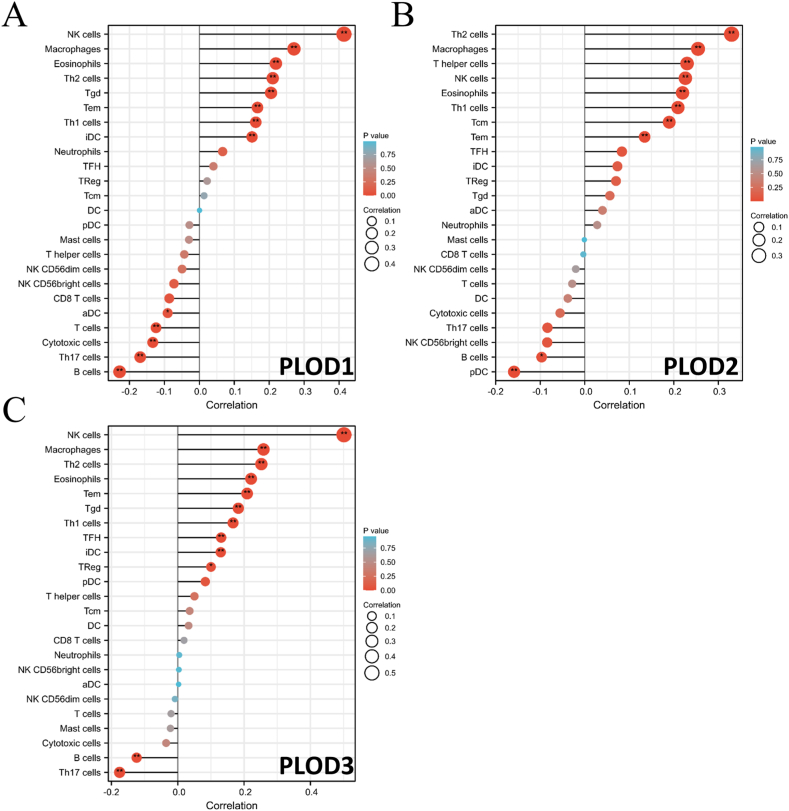
Single sample gene set enrichment analysis (ssGSEA) for various PLODs in Head and Neck Squamous Cell Carcinoma (HNSCC). **(A)** PLOD1; **(B)** PLOD2; **(C)** PLOD3. *p < 0.05 **p < 0.01.

The next set of evaluations revealed that increased PLOD expression was linked to an increased Stromal (Fig. 4A–C, all p < 0.001) and ESTIMATEScore (Fig. 4A–C, all p < 0.01). In addition, the expression of all three PLODs were positively correlated with increased immune infiltration with these samples presenting with increased numbers of CD4^+^ T cells, macrophages, neutrophils, and dendritic cells (Supplementary Figure S1, all p < 0.01). SsGSEA also showed that the expression of all three PLOD was positively correlated with NK cell, macrophage, eosinophil, Th2 cell, T effector memory (Tem) cell, and Th1 cell infiltration (Fig. 5A–C, all p < 0.01). Additionally, PLOD1 and PLOD3 expression were positively correlated with the expression of T gamma delta (Tgd) cells and immature DC (iDC) cells, while PLOD2 was not. These evaluations also revealed that the expression of all three PLOD family members was negatively correlated with B cell infiltration in these samples (Fig. 5A–C, all p < 0.01).

### 3.5 Gene set enrichment analysis for PLOD genes in HNSCC

**Fig. 6:**
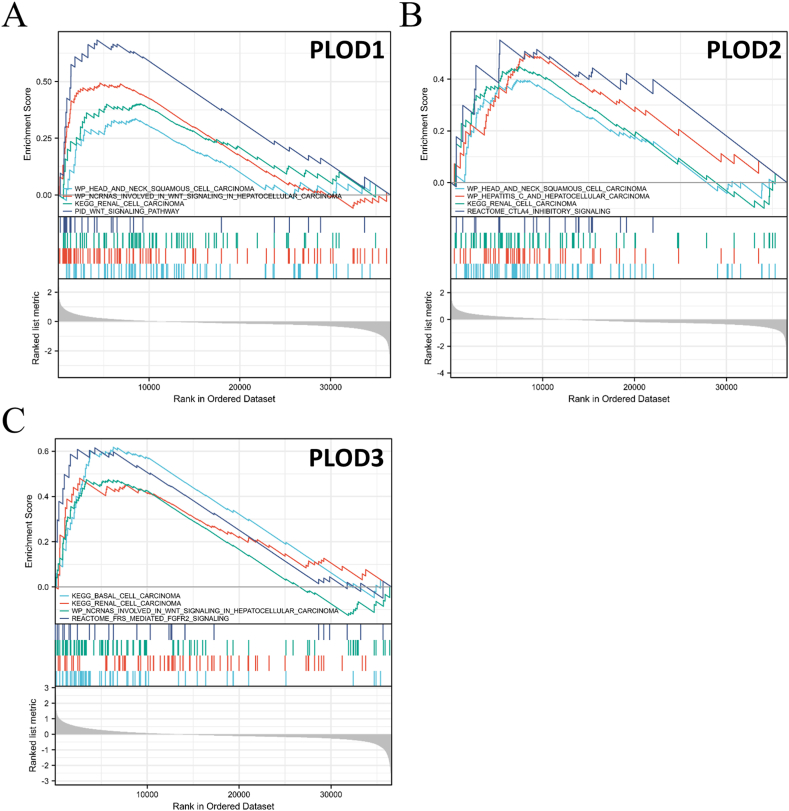
Gene set enrichment analysis (GSEA) describing the relationship of various PLODs with different kinds of tumors and signal transduction pathways. **(A)** PLOD1; **(B)** PLOD2; **(C)** PLOD3.

Gene set enrichment analysis (GSEA) was then used to explore the potential biological processes, signal transduction pathways, and potential tumor types associated with HNSCC progression. These evaluations demonstrated that PLOD1 is linked to HNSCC and the ncRNAs involved in WNT signaling in hepatocellular carcinoma, renal cell carcinoma, and various other signaling pathways (Fig. 6A). PLOD2 was related to HNSCC, hepatitis C, hepatocellular carcinoma, renal cell carcinoma, and CTLA4 inhibitory signaling (Fig. 6B) while PLOD3 was linked to basal cell carcinoma, renal cell carcinoma, ncRNAs involved in WNT signaling in hepatocellular carcinoma, and FRS-mediated FGFR2 signaling (Fig. 6C).

## 4 Discussion

Previous studies have shown that PLOD3 expression is linked to poor prognosis and immune cell infiltration in HNSCC [21]. Here, our systematic analysis of the association between PLOD expression and HNSCC was performed using data from the TCGA, GEO and GTEx databases. PLOD family members (PLOD1, PLOD2, and PLOD3) were shown to be associated with poor prognosis and could act as potential biomarkers or therapeutic targets for both gastric cancer and hepatocellular carcinoma [18,20,22]. Our previous studies indicated that PLOD was also associated with lower grade glioma and soft tissue sarcoma [22,23]. Deeper analysis showed that PLOD2 may be a crucial regulator in the invasion or metastasis of HNSCC [30]. Here, we identified that all three members of the PLOD family are abundantly expressed in tumor tissues and could act as potential biomarkers for HNSCC.

PLODs encode the lysyl hydroxylase (LH) protein, which is involved in the biosynthesis of collagen. Therefore, abnormal or altered expression of PLODs could be a potential factor in the pathogenesis of some diseases [31,32]. This could be the result of various factors including the induction of epithelial–mesenchymal transition (EMT) in response to PLOD overexpression. This is because when the disordered tissues are repaired, abundant expression of the PLODs could instigate EMT. During EMT, epithelial cells may adopt an unsteady state; therefore, losing polarity and adhesion to their original position [33,34]. EMT is essential in normal processes, such as wound healing but may also contribute to tumor formation [35]. Consequently, the overexpression of PLODs could be associated with EMT, and this might help the tumor grow and acquire the ability to invade the surrounding tissues. In addition, the TME may also play a crucial role in the relationship between PLOD expression and HNSCC. PLODs impact collagen expression, which is an essential component of the ECM and occupies most of the TME [16,17,36]. LH is involved in collagen cross-linking within the ECM thus changes in this cross-linking can change the supply of the chemical and physical substructures needed for cancer formation and proliferation [17,37]. Consequently, dysregulation of PLODs could be associated with the proliferation of various tumors, including glioma, gastric, and lung cancer [[18], [19], [20],^38^]. In addition, our previous study identified that PLODs could be regulators in lower-grade gliomas and soft tissue sarcoma, which could further support this hypothesis [22,23]. The third possible mechanism linking PLODs to HNSCC is the Wnt signaling pathway. GSEA analysis revealed that PLOD1 and PLOD3 were linked to the Wnt signaling pathway. Previous studies also support the hypothesis that the Wnt signaling pathway may play an essential role in tumorigenesis and invasion in HNSCC [39,40]. In addition, other studies have shown that the Wnt signaling pathway could be a potential target for the treatment of HNSCC [41,42]. Interestingly, our GSEA analysis showed that the PLODs were associated with both HNSCC and liver cancer and revealed novel connections to basal cell carcinoma and renal cell carcinoma, which have not been previously reported. Thus, the relationship between PLODs and these additional cancers is worthy of analysis.

This study evaluated the expression of PLODs in HNSCC using data from the TCGA, GEO and GTEx cohorts. These studies revealed that the PLODs were abundantly expressed in HNSCC tumors when compared to the corresponding normal tissues. Although the finding was not validated by experiment, this finding matched our previous observations demonstrating that PLODs were highly expressed and positively correlated in lower grade glioma tissues (LGG) [22]. Therefore, PLODs may not only be oncogenes in HNSCC, but also in some other tumors, such as LGG. However, the analysis of the effects of combined PLOD (PLOD1, PLOD2, and PLOD3) expression remains poor. Additionally, PLOD1 and PLOD3 expression was linked to poor prognosis in OS, DSS, and PFS, while PLOD2 expression was only linked to poor prognosis in OS and DSS based on TCGA database. A long-term follow up analysis could be further confirmed this finding. DSS and PFS are also regarded as indices for the evaluation of new treatments or drugs [43,44]. Thus, these results may indicate that PLODs could be novel targets for clinical therapies targeting HNSCC.

PLOD overexpression could be closely associated with HNSCC; therefore, supplementary analysis of the PLOD-related genes was also completed in an effort to identify the likely interactions underlying HNSCC pathogenesis. A total of nine genes were linked to all three PLODs (PLOD1, PLOD2, and PLOD3) and all nine of these genes were positively correlated with other critical genes associated with HNSCC, suggesting that they may also act as oncogenes in HNSCC. Given this, it is not surprising that some of these genes were associated with HNSCC and may even be linked to poor prognosis in these patients. ITGA5 is correlated with poor prognosis and may lead to the proliferation or invasion of HNSCC making it a clear potential target for HNSCC therapy [45,46]. Previous studies have also shown that MMP14 is highly expressed in tongue squamous cell carcinoma (TSCC) and that this protein may be a target for reducing migration and invasion in TSCC via its regulation of miR-34a, a well-known tumor suppressor gene [47]. Moreover, GALNT2 could also be a regulator of HNSCC because it may enhance the migration and invasion of HNSCC [48]. Furthermore, the combination of P4HA1, PLOD1, and PLOD2 has been previously reported as an important signature for cycle regulation and cell adhesion maintenance, which may explain the poor prognosis in HNSCC [49]. The combination of PLOD2 and P4HA1 may also be related to tumorigenesis in a mouse model [50]. Previous studies have also shown that P4HA1 is linked to poor prognosis and is a potential therapeutic target for HNSCC [51,52]. Consequently, we suppose that the PLODs and PLOD-related genes are potential biomarkers and therapeutic targets for HNSCC and thus worthy of further analysis.

We also found a correlation between PLOD expression and immune infiltration. In vitro evaluations suggest that high expression of the PLOD genescould be associated with increased Stromal- and ESTIMATEScore, which means that the overexpression of PLODs is likely linked to the high level of stromal cell infiltration and the lower purity of tumor cells in HNSCC [29]. SsGSEA analysis was employed to explore the abundance of immune cells. PLOD expression was positively correlated with the increased abundance of some immune and immunostimulatory cells, such as natural killer cells and type 1 T helper cells, and negatively correlated with some immunosuppressive cells, such as macrophages and type 1 T helper cells [53,54]. This finding worth to be further confirmed by in vivo or in vitro experiment. Consequently, individuals with increased PLOD expression may experience a “hot” but suppressive immune environment within the TME, indicating that PLODs might be critical regulators of the TME in HNSCC. Thus, PLODs may be promising therapeutic targets for improving the prognosis and response to anti-tumor treatment in HNSCC [55,56].

However, there are still some limitations in this study. The TCGA data could be affected by some factors which may be involved in the regulation of gene expression. Experiments base on in vivo or in vitro models could make our results more reliable and we would put our efforts on this field in the future.

## 5 Conclusion

In conclusion, this study demonstrates the potential role of PLODs in HNSCC tumor formation and invasion. This indicates that overexpression of PLODs may result in tumorigenesis in HNSCC. PLODs may be promising therapeutic targets in HNSCC. Consequently, PLODs may act as potential biomarkers for the personalized treatment of HNSCC in the future.

## Author contributions

Conceived and designed the experiments: Siming Gong, Changwu Wu, Yingjuan Duan, Yuling Wang, Juyu Tang and Panfeng Wu.

Performed the experiments: Siming Gong, Yuling Wang, Hao Wu and Bixi Zhang.

Analyzed and interpreted the data: Siming Gong, Changwu Wu, Hao Wu and Bixi Zhang.

Contributed reagents, materials, analysis tools or data: Siming Gong, Changwu Wu, Hao Wu and Bixi Zhang.

Wrote the paper: Siming Gong, Changwu Wu and Panfeng Wu.

All authors approved the final manuscript and accounted for all aspects of work, read, and agreed to the published version of the manuscript.

## Funding

This research was funded by 10.13039/501100001809National Natural Science Foundation of China, grant number 82072194.

## Declaration of competing interest

The authors declare that they have no known competing financial interests or personal relationships that could have appeared to influence the work reported in this paper.
