# Supplementary material for: PLODs: Novel prognostic biomarkers and potential immunotherapy targets for head and neck squamous cell carcinoma
Source: Heliyon. 2023 Feb 3;9(2):e13479. doi: 10.1016/j.heliyon.2023.e13479 (PMC9937984; doi:10.1016/j.heliyon.2023.e13479)
Supplement: Multimedia component 4 [file mmc4.docx]

**Supplementary Table S1.** The overview of HNSCC patients.

| Characteristic | levels | Overall |
| --- | --- | --- |
| n |  | 502 |
| T stage, n | T1 | 33 |
|  | T2 | 144 |
|  | T3 | 131 |
|  | T4 | 179 |
| N stage, n | N0 | 239 |
|  | N1 | 80 |
|  | N2 | 154 |
|  | N3 | 7 |
| M stage, n | M0 | 472 |
|  | M1 | 5 |
| Clinical stage, n | Stage I | 19 |
|  | Stage II | 95 |
|  | Stage III | 102 |
|  | Stage IV | 272 |
| Radiation therapy, n | No | 154 |
|  | Yes | 287 |
| Gender, n | Female | 134 |
|  | Male | 368 |
| Histologic grade, n | G1 | 62 |
|  | G2 | 300 |
|  | G3 | 119 |
|  | G4 | 2 |
| Anatomic neoplasm subdivision, n | Alveolar Ridge | 18 |
|  | Base of tongue | 23 |
|  | Buccal Mucosa | 22 |
|  | Floor of mouth | 61 |
|  | Hard Palate | 7 |
|  | Hypopharynx | 10 |
|  | Larynx | 111 |
|  | Lip | 3 |
|  | Oral Cavity | 72 |
|  | Oral Tongue | 126 |
|  | Oropharynx | 9 |
|  | Tonsil | 40 |
| Age, n | <=60 | 245 |
|  | >60 | 256 |


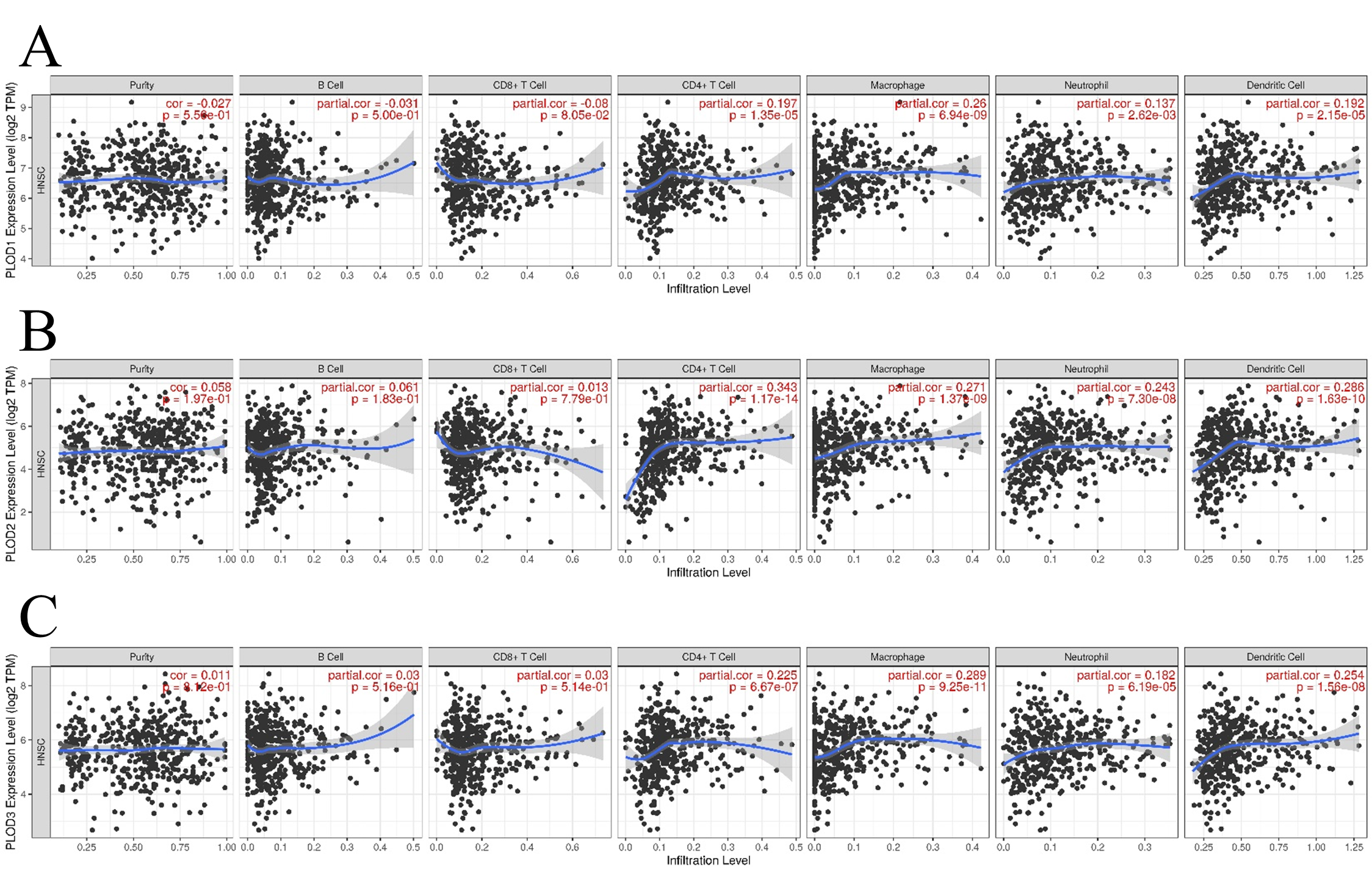


**Supplementary Figure S1.** Correlations of PLOD family members expression with immune cells infiltration level in HNSCC. **(A)** PLOD1 **(B)** PLOD2 **(C)** PLOD3
